# Light Down-Converter Based on Luminescent Nanofibers from the Blending of Conjugated Rod-Coil Block Copolymers and Perovskite through Electrospinning

**DOI:** 10.3390/polym12010084

**Published:** 2020-01-03

**Authors:** Dai-Hua Jiang, Saburo Kobayashi, Chih-Chun Jao, Yoshinobu Mato, Takuya Isono, Yu-Han Fang, Chun-Che Lin, Toshifumi Satoh, Shih-Huang Tung, Chi-Ching Kuo

**Affiliations:** 1 Institute of Organic and Polymeric Materials, Research and Development Center of Smart Textile Technology, National Taipei University of Technology, Taipei 10608, Taiwan; d06549013@ntu.edu.tw (D.-H.J.); a405292005@gmail.com (C.-C.J.); au102324018@gmail.com (Y.-H.F.); cclin0530@gmail.com (C.-C.L.); 2 Institute of Polymer Science and Engineering, National Taiwan University, Taipei 106, Taiwan; 3 Chemical Sciences and Engineering, Hokkaido University, Sapporo 060-8628, Japan; saburou_k@eis.hokudai.ac.jp (S.K.); mato@eis.hokudai.ac.jp (Y.M.); 4 Faculty of Engineering, Hokkaido University, Sapporo 060-8628, Japan; isono.t@eng.hokudai.ac.jp

**Keywords:** rod/coil block copolymer, perovskite, polymer, electrospinning, light down-converter

## Abstract

We demonstrated a novel strategy for the preparation of light down-converter by combining rod-coil block copolymers with perovskite quantum dots (QDs) through electrospinning. Reports have shown that polymer deformability can be enhanced by incorporating a soft segment and controlled by varying the rod/coil ratio. Therefore, we first synthesized the rod-coil block copolymer through the click reaction of polyfluorene (PF) and poly(*n*-butyl acrylate) (PBA). Next, the CsPbBr_3_@PF_8k_-*b*-PBA_12k_ composite fibers were fabricated by blending perovskite through electrospinning. Optical spectral evidence demonstrated the success of the strategy, as light down-converters were prepared through the controlled variance of QD/polymer ratios to achieve tunable color and stretchability. This result reveals the potential of using rod-coil block copolymers to fabricate color-tunable perovskite light down-converters.

## 1. Introduction

Several organic semiconducting materials have been studied due to their potential beneficial properties, such as solution processability [1,2], band gap tuning [3,4,5], chemical structure, and self-assembly [6,7]. Satisfactory intrinsic active materials must be able to sustain large mechanical deformation and maintain high semiconducting properties for use in stretchable optical devices due to the growth of artificial intelligence technology [8,9,10,11]. With the growing enthusiasm for bioinspired robotics and healthcare wearables, it is essential to develop materials that can be flexible under high deformation and adhere to irregular surfaces [12,13,14,15]. Therefore, conjugated polymers incorporating elastomeric materials provide promising opportunities for satisfying current requirements. Several methods, including polymer blending [16,17,18,19], embedding [20,21], and chemical modification [22,23], have been widely utilized to enhance the stretchability and flexibility of materials. For example, Bao et al. [24] revealed a potential material based on 3, 6-di(thiophen-2-yl)-2,5-dihydropyrrolo [3,4-c] pyrrole-1,4-dione (DPP) backbone, which could maintain high field-effect mobility (1.12 cm^2^ V^−1^ s^−1^) under 100% strain. It can fully recover after damage via a solvent and thermal healing treatment due to network cross-linking with hydrogen bonding. Furthermore, the polymer structure strongly affects the device performance in the solid-state film; thus, rod-coil copolymerization based on a conjugated polymer with elastomeric polymer is considered a promising approach. Subsequently, our lab developed a series of block copolymers, such as: poly(3-hexylthiophene)-block-poly(*n*-butyl acrylate) (P3HT-*b*-PBA) and polyfluorene-block-poly(pendent isoindigo) (PF-*b*-Piso) for stretchable field-effect transistor and resistive memory applications [25,26]. In both of the cited studies, the materials were designed and synthesized through the copper-catalyzed azido-alkyne click reaction, which is widely used for the synthesis of block copolymers. The studies have shown that electronic properties and interchain organizations could be tuned by varying the rod/coil ratio [27,28]. However, the studies have mainly focused on stretchable field-effect transistors and memory devices [28,29], whereas stretchable light-emitting materials have received less attention.

Polyfluorenes (PFs) with different unit copolymers have been extensively used for efficient light-emitting diodes (LEDs) or light down-converters. This is because it is well-known for having blue-emitting conjugated moiety with inherent advantages, such as efficiency hole carrier transport, color tuning, effective thermal ability, and outstanding photoluminescence quantum yields (PLQYs) [30,31]. Thus, PF-based block copolymers are more beneficial for producing organic optoelectronic devices [32,33]. The strong *π-π* stacking interchain interaction attributed to conjugated chains can be the driving force behind the formation of self-assembled nanostructures with crystalline order. This could affect the charge transport properties and performance of optoelectronic devices [27]. Recent studies have used fluorene copolymer for elucidating changes of packing structure and affecting fluorene structure in terms of optoelectronic and charge transport properties by different annealed states of different units [34,35]. Mo et al. utilized PF homopolymer derivative and carbazole grafting silafluorene for blue LEDs and revealed high external quantum efficiency (4.1%) and high-quality deep blue emissions [CIE coordinates (0.16, 0.08)] [34,35]. However, the major drawback of utilizing homopolymer PF is its vulnerability when stretched due to its rigidity. To address this problem, our lab incorporated soft segments into block copolymers to enhance their stretchability. The thin film, consisting of poly [2, 7-(9, 9-dihexylfluorene)]-block-poly(*n*-butyl acrylate) (PF-*b*-PBA), could form obvious self-assembled nanofibrillar structures after solvent annealing treatment [27]. Moreover, the stretchability and optical properties of the materials were controllable by adjusting the rod/coil segment ratio. However, the optical and device performance of such designed materials decreased upon a coil ratio increase; this was due to a decrease in the conjugated phase domains [25,26]. Therefore, intrinsically stretchable light-emitting materials with high device performance that can operate under high mechanical strain, are necessary for the future.

Versatile soft materials have been combined with perovskite in stretchable LED devices or light down-converters [36,37,38]. For example, Mandal et al. reported on methylammonium lead bromide (CH_3_NH_3_PbBr_3_) (MAPbBr) being introduced to poly (vinylidene fluoride) (PVDF) nanofiber through electrospinning. Electroactive phase composition can enhance the output power and tensile strength of the nanofiber mats [39]. In our previous work, composite CsPbX_3_ (X = Cl, Br, and I) perovskite nanocrystals (NCs) were encapsulated with stretchable [poly(styrene-butadiene-styrene); SBS] fibers by electrospinning on light down-converters. This was maintained for longer than 1 h in water and the material was stretched under 170% strain without obvious cracks showing; furthermore, the LED chip device still maintained high luminance and performance under low voltage [38]. Numerous studies have aimed to discover novel approaches for fabricating a white light-emitter with perovskite [40,41]. Fabrication processes for different colors of perovskite could even be implemented by adjusting halide components. The white light-emitter based on perovskite still needs to be used in full-color displays [38,42,43,44]. Therefore, multilayer by different colors for applying to white light-emitter is the conventional strategy but is complicated and troublesome. Encouraged by previous reports, in this paper we report on stretchable and blue light-emitter rod-coil di-block copolymers (PF-*b*-PBA) blended with perovskite (CsPbBr_3_) to make nanofiber membranes through electrospinning on light down-converters. The rod-coil di-block copolymers (PF-*b*-PBA) were synthesized through the click reaction with ethynyl-terminated PF and azido-terminated PBA homopolymers, in accordance with a previous study [27]. To increase the viscosity of the di-block copolymers, which facilitates electrospinning, we synthesized PF-*b*-PBA with high molecular weight by combining Suzuki-Miyaura coupling polymerization for the PF block and atom transfer radical polymerization (ATRP) for the PBA block. Owing to the multifunction of electrospinning, we applied on an LED chip by using a nanofiber mat, which we fabricated via electrospinning [45,46,47,48,49,50,51]. The effects of the nanofiber mat prepared through electrospinning from PF-*b*-PBA blended with perovskite (CsPbBr_3_) were analyzed for deformation morphology and optical properties by using optical microscopy, UV-visible spectrometry, spectrofluorometry (PL), and confocal microscopy. In addition, we employed electrospinning to develop white-light-emitting fibers that can be color tuned by varying the perovskite/PF ratio. This work represents an authoritative stride in the field of perovskite electronics by opening new possibilities for white-light-emitting down-converter devices.

## 2. Results and Discussion

**Scheme 1:**
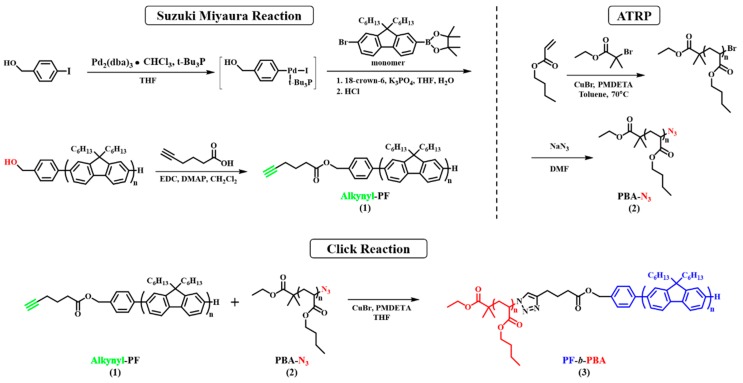
Synthetic route for PF-*b*-PBA rod-coil block copolymer.

**Figure 1:**
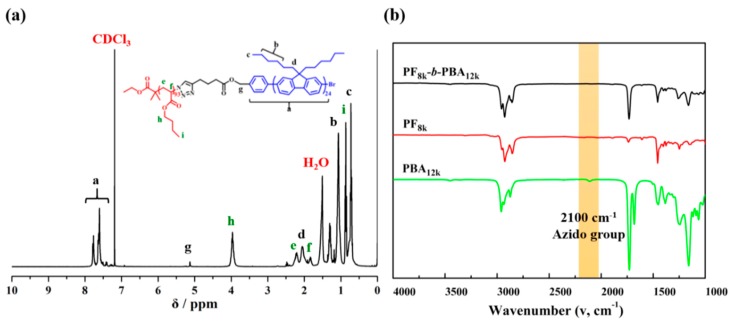
(**a**) ^1^H-NMR spectrum of the PF_8k_-*b*-PBA_12k_ in CDCl_3_ and (**b**) FTIR spectra of the PF_8k_, PBA_12k_-N_3_ and PF_8k_-*b*-PBA_12k_ copolymers.

To prepare rod-coil block copolymers via the copper-catalyzed click reaction, first, the homopolymer with appropriate functional groups were synthesized in accordance with previous reports [31]. A benzyl alcohol-terminated polyfluorene (PF_8k_-BnOH) was synthesized using Suzuki-Miyaura coupling polymerization and then modified to the alkynyl-terminated PF (1, alkyne-PF_8k_) through esterification with 5-hexynoic acid. The ^1^H NMR spectra of the PF_8k_-BnOH and PF_8k_-C≡CH clearly show the shift in the benzyl proton (from 4.80 ppm to 5.24 ppm) and appearance of new signals due to the hexynoate moiety (f: 2.55, h: 2.32, i: 2.00, and g: 1.92 ppm) after the modification, which indicated the successful synthesis of PF_8k_-C≡CH (Figure S1). The azido-terminated PBA was produced through atom transfer radical polymerization (ATRP) using 2-bromo-2methylpropanoate as the initiator. Subsequently, sodium azide was utilized to convert the bromo-terminated group to the azido group. The ^1^H NMR spectra of azido-terminated PBA (2, PBA-N_3_) backbone (a: 1.82–1.95 and b: 2.15–2.41 ppm) and butyl side chain (c: 3.89–4.08, d and e: 1.45–1.65, and f: 0.85–0.97 ppm) are depicted in Figure S2. The structures of the PBA were further confirmed by the FTIR spectrum (Figure S3). The FTIR spectrum of the PBA displayed a characteristic stretching band at 2100 cm^−1^, which corresponds to the azido group. Finally, the rod-coil block copolymer, poly [2, 7-(9, 9-dihexyl-fluorene)]-*block*-poly(*n*-butyl acrylate) (PF-*b*-PBA), was synthesized by coupling ethynyl-terminated PF with azido-terminated PBA; the synthetic pathway is illustrated in Scheme 1. The molecular characteristics of polymers are listed in Table 1. Similarly, the ^1^H NMR signals of PF_8k_-*b*-PBA_12k_ exhibited the same characteristic protons of long alkyl chain from both the PF and PBA segments, and the peak at 2100 cm^−1^ due to presence of the azido group completely disappeared from the FTIR spectrum (Figure 1). In addition, the SEC traces of the polymers (Figure S4) displayed a unimodal peak with the *M*_w_/*M*_n_ value of 1.43, which clearly shifted toward a higher molecular weight region compared with that of the homopolymer owing to the successful coupling reaction.

Next, we investigated the thermal behavior of the polymers by using TGA and DSC; the results are summarized in Table 2. We reveal that the thermal degradation temperature (*T_d_*, 95% weight loss) of the PF_8k_-*b*-PBA_12k_ (350 °C) is in between the PF_8k_ (401 °C) and PBA_12k_-N_3_ (340 °C) homopolymers, which shows favorable thermal stability(Figure S5). Moreover, the PF_8k_-*b*-PBA_12k_ exhibited two glass transition temperatures (*T_g_*) that are from individual homopolymers: 78 °C (PF_8k_) and −54 °C (PBA_12k_-N_3_). The individual phase transitions from PF and PBA are due to the incompatibility between the rod PF and the coil PBA phase; these tend to separate into different domains, resulting in the presence of two *T_g_* values [27].

**Figure 2:**
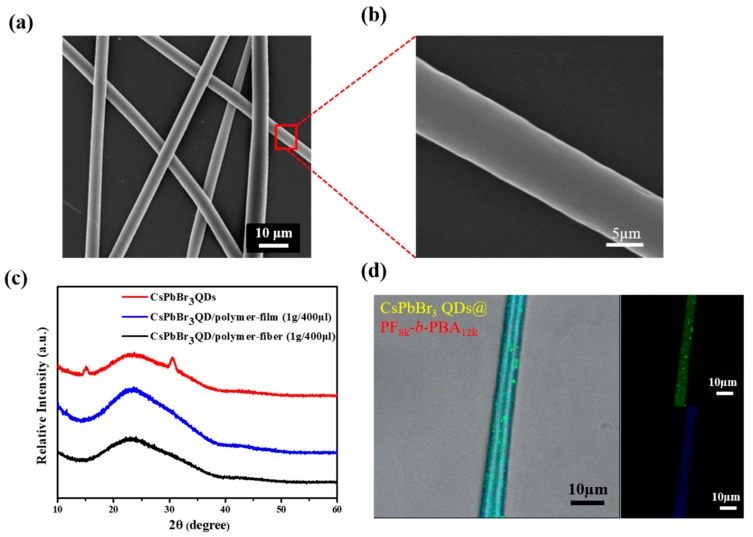
(**a**,**b**) SEM images of the CsPbBr_3_@PF_8k_-*b*-PBA_12k_ fibers (1 g/400 μL). (**c**) XRD patterns of the CsPbBr_3_@PF_8k_-*b*-PBA_12k_ fiber compared with CsPbBr_3_@PF_8k_-*b*-PBA_12k_ and CsPbBr_3_ QD film. (**d**) Confocal image of the CsPbBr_3_ QDs@PF_8k_-*b*-PBA_12k_ fibers.

The SEM images of CsPbBr_3_ QDs@PF_8k_-*b*-PBA_12k_ composite fibers (1 g block copolymer and 400 μL perovskite dissolve in 2 mL CH_2_Cl_2_) prepared by electrospinning are presented in Figure 2a,b. The fabricated fibers are uniform and show average diameters of 8–10 μm. Moreover, their surface morphology is smooth, indicating that no perovskite crystals appeared on the fiber surface. Figure 2c shows the XRD data of the CsPbBr_3_@PF_8k_-*b*-PBA_12k_ fiber (1 g/400 μL) compared with CsPbBr_3_@PF_8k_-*b*-PBA_12k_ (1 g/400 μL) and CsPbBr_3_ QDs films. The CsPbBr_3_@PF_8k_-*b*-PBA_12k_ film and CsPbBr_3_@PF_8k_-*b*-PBA_12k_ fiber did not show any perovskite peak. We speculate that this is due to theinside of the fiber. Therefore, we conducted confocal imaging for CsPbBr_3_ QDs@PF_8k_-*b*-PBA_12k_ that was excited by ultraviolet (UV) light at a wavelength of 380 nm, as shown in Figure 2d. The perovskite and the PF in the sample emitted their original light colors, which verified that they retained their fluorescent properties after electrospinning and that the perovskite QDs were encapsulated inside the polymer fiber. The UV-vis absorption and PL emission of the polymers prepared by spin coating and electrospinning are summarized in Table 2. The photophysical property of their corresponding solution is presented in Figure S6. In the case of the as-cast film, the λ^abs^_max_ values of PF_8k_ and PF_8k_-*b*-PBA_12k_ were observed at 384 and 386 nm due to the π-π conjugation of the PF segment. Concerning the as-cast film of λ^PL^_max_ values, we can clearly observe the blueshift from PF_8k_-*b*-PBA_12k_, a phenomenon which could explain the phase separation from the PF and PBA segment. The blueshift was due to the aggregation of the PF phase. For the as-cast film of CsPBr_3_ @PF_8k_-*b*-PBA_12k_, the π-π conjugation peak was also observed at 382 nm, and the λ^PL^_max_ values showed peaks at 445, 471, and 510 nm, respectively. The distinct peaks of 445 and 471 nm relate to the different crystal phases of the PF and the peak of 510 nm is from the CsPbBr_3_ QDs.

**Figure 3:**
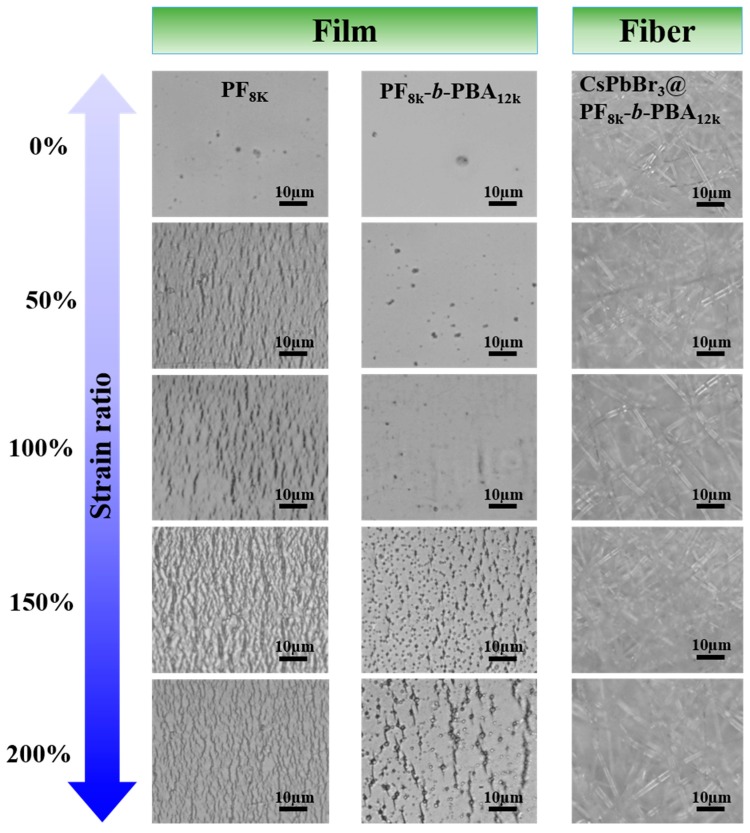
The crack-onset test for the PF_8k_ and PF_8k_-*b*-PBA_12k_ films and CsPbBr_3_ @PF_8k_-*b*-PBA_12k_ composite fibers (1 g/400 μL).

The deformation of the PF homopolymer, PF_8k_-*b*-PBA_12k_ block copolymer film, and CsPbBr_3_ QDs@PF_8k_-*b*-PBA_12k_ composite fibers (1 g/400 μL) were measured directly using an optical microscope under different strains, as shown in Figure 3. The polymer samples were prepared onto the polyurethane (PU) substrate by spin coating and electrospinning. All unstrained samples were observed without any cracks. By contrast, in the PF_8k_ homopolymer sample, the cracks continuously enlarged as the strain increased. Due to the incorporation of the PBA soft segment, the stretchability of PF_8k_-*b*-PBA_12k_ block copolymer was clearly enhanced. In addition, the stretchability of CsPbBr_3_ QDs@PF_8k_-*b*-PBA_12k_ composite fibers exhibited good deformability even under 100% strain. However, the PF_8k_-*b*-PBA_12k_ film started to crack when the strain reached 150%. By contrast, the CsPbBr_3_ QDs@PF_8k_-*b*-PBA_12k_ fiber membrane exhibited no cracks even under 200% strain. This confirms that coil segment modification and nano-scale change by electrospinning can effectively enhance the stretchability of material [52,53].

**Figure 4:**
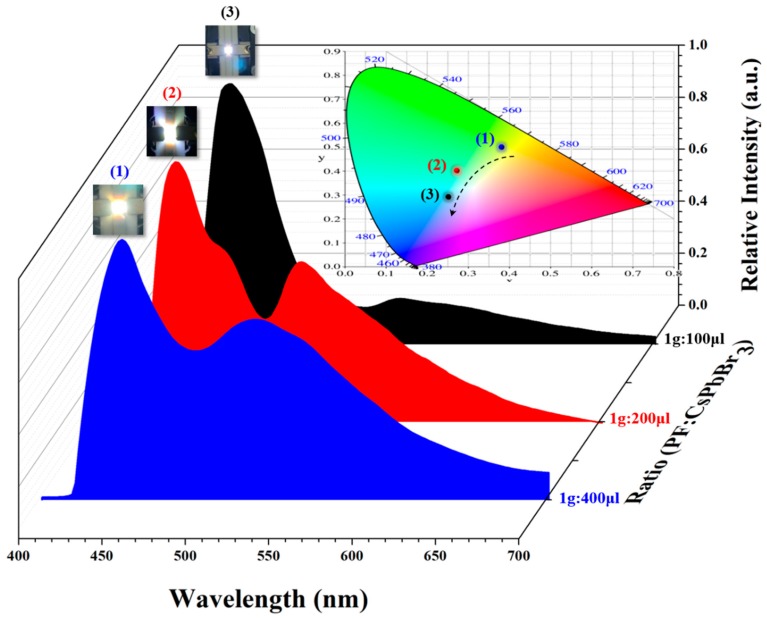
Emission spectra of different color light down-converter with CIE color coordinates obtained using CsPbBr_3_@ PF_8k_-*b*-PBA_12k_ composite fibers under UV chip (380 nm) excitation and an applied voltage of 3 V. (Inset images show the light down-converter with an applied QD/polymer composite fiber ratio of 100:1, 200:1, and 400:1 (μL/g)).

The color-tunable light down-converters were successfully fabricated using a single layer of CsPbBr_3_@PF_8k_-*b*-PBA_12k_ composite fiber on a commercial UV chip (λ_max_ = 380 nm) and commission internationale de l’eclairage (CIE) is illustrated in Figure 4. Furthermore, the color of the light down-converter can be controlled by varying the QD/polymer ratio by 1:100, 1:200, and 1:400 (1 g/400 μL) (Figure 4). The insets in Figure 4 display the light down-converter with different correlated color temperatures (CCTs), which correspond to the emission images. The emissive intensity of perovskite becomes weaker when the ratio decreases. In other words, the higher intensity at 550 nm on green-color-emission is observed with an increasing perovskite QD blending ratio. It is because of the obvious energy transfer (physical processes) from commercial UV LED chip (donor) and PF-block (donor) to perovskite QD (acceptor), which is similar to some papers [38,44]. In this way, the CCTs and color can turn from green to blue. It is a novel and simple method of controlling the color by blending perovskite in the polymer fiber.

## 3. Conclusions

We successfully synthesized rod-coil PF_8k_-*b*-PBA_12k_ block copolymers. Through the incorporation of a PBA soft segment, such a block copolymer is able to maintain high stretchability, which facilitates its application in a variety of functions, such as an electron-hole transport layer or emission layer in light down-converters. Furthermore, we utilized a simple electrospinning method to combine conjugated block copolymer with perovskite QDs to prepared light down-converters by single-layer fiber mats. In contrast to the multicolor stack and perovskite’s halogen substitute methods, the light down-converter color can be tuned by varying the QD/polymer ratios and only using a single layer due to the double fluorescence combination. The results of the present study suggest that block copolymers combined with perovskite have the potential to achieve high stretchability as well as favorable fluorescent properties for versatile applications that require outstanding optical properties.

## Figures and Tables

**Table 1:** Molecular characteristics of the PF_8k_, PBA_12k_-N_3_ and PF_8k_-*b*-PBA_12k_ copolymers.

Sample	PBA *^a^* (wt%)	*M*_n,NMR_ *^a^* (g mol^−1^)	*M*_n,SEC_ *^b^* (g mol^−1^)	*M*_w_/*M*_n_ *^b^*
PF_8k_	0	8200	9400	1.38
PBA_12k_-N_3_	100	11,700	12,500	1.10
PF_8k_-*b*-PBA_12k_	72	19,100	21,400	1.43

*^a^* Calculated from ^1^H NMR spectra of the polymers in CDCl_3_. *^b^* Determined by SEC in. THF using polystyrene standard.

**Table 2:** Physical properties of the PF_8k_, PBA_12k_-N_3_, PF_8k_-*b*-PBA_12k_ and CsPbBr_3_ @PF_8k_-*b*-PBA_12k_ (1 g/400 μL).

Sample	*T_d_* (°C)	*T_g_* (°C)	As-Cast Film		Nanofiber Membrane
			λ^abs^_max_ (nm)	λ^PL^_max_ (nm)	λ^PL^_max_ (nm)
PF_8k_	401	78	384	451,481	--
PBA_12k_	340	−54	--	--	--
PF_8k_-*b*-PBA_12k_	350	−78,110	386	445,466	446
CsPbBr_3_@PF_8k_-*b*-PBA_12k_	--	--	382	445,471,510	446,518

## Supplementary Materials

The following are available online at https://www.mdpi.com/2073-4360/12/1/84/s1. ^1^H-NMR spectra of the polymers; FTIR of the PBA homopoymers; SEC profiles of the polymers; TGA curves, UV-vis absorption, and PL emission spectrum of the polymers.
