# Supplementary material for: Light Down-Converter Based on Luminescent Nanofibers from the Blending of Conjugated Rod-Coil Block Copolymers and Perovskite through Electrospinning
Source: Polymers (Basel). 2020 Jan 3;12(1):84. doi: 10.3390/polym12010084 (PMC7023616; doi:10.3390/polym12010084)
Supplement: Supplementary file 1 [file polymers-12-00084-s001.pdf]

Supporting Information

Light Down-Converter Based on Luminescent Nanofibers from the Blending of Conjugated Rod-Coil Block Copolymers and Perovskite through Electrospinning

Dai-Hua Jiang,[a,b,c] Saburo Kobayashi,[c] Chih-Chun Jao,[a] Yoshinobu Mato,[c] Takuya Isono,[d] Yu-Han Fang,[a] Chun-Che Lin,[a] Toshifumi Satoh,[d,*] Shih-Huang Tung,[b,*] and Chi-Ching Kuo,[a,*]

[a] Institute of Organic and Polymeric Materials, Research and Development Center of Smart Textile Technology, National Taipei University of Technology, 10608 Taipei, Taiwan.

[b] Institute of Polymer Science and Engineering, National Taiwan University, 106 Taipei, Taiwan.

[c] Chemical Sciences and Engineering, Hokkaido University, Sapporo 060-8628, Japan

[d] Faculty of Engineering, Hokkaido University, Sapporo 060-8628, Japan

*Author to whom all correspondence should be addressed Tel.: 886-2-27712171 (ext. 2407); Fax: 886-2-27317174

Correspondence to: *Prof. C.-C. Kuo kuocc@mail.ntut.edu.tw; *Prof. S.-H. Tung shtung@ntu.edu.tw; *Prof. T. Satoh satoh@eng.hokudai.ac.jp

1. Materials and Methods

1.1. Materials

2-(7-Bromo-9, 9-dihexyl-9H-fluorene-2-yl) 4, 4, 5, 5-tetramethyl-1, 2, 3-dioxaborolane were prepared according to the reported literature studies. [1,2] Tris-(dibenzylideneacetone)palladium ($Pd_2(dba)_3$, >97%), ethyl 2-bromo-2-methylpropanoate (≥98%), copper(I) bromide (CuBr, >98%), sodium azide (NaN_3, >99%), tetrahydrofuran (THF, anhydrous, ≥99%), methanol (MeOH, ≥99%), dichloromethane (CH_2Cl_2, anhydrous, ≥99%), toluene (anhydrous, ≥99%), chloroform ($CHCl_3$, anhydrous, ≥99%), *N, N*-dimethylformamide (DMF, ≥99%), oleylamine (primary amine, >98%), oleic acid (90%), cesium carbonate (99.995%), 1-octadecene (90%), and lead (ll) bromide were purchased from Sigma-Aldrich, Saimt Louis, USA. Tri (*tert*-butyl)phosphine (t-Bu3P, >96%) and tripotassium phosphate (K_3PO_4, >95%) were obtained from Wako Pure Chemical, Osaka, Japan. 4-iodobenzyl alcohol (>99.0%), 5-hexynoic acid (>95%), 18-crown-6 (>98%), *n*-butyl acrylate (BA, >99%), 1-ethyl-3-(3-dimethylaminopropyl) carbodiimide (EDC, >98%), 4-dimethylaminopyridine (DMAP, >99%), and *N, N, N´, N″, N″*-pentamethyldiethylenetriamine (PMDETA, >99%) were bought from Tokyo Chemical, Tokyo, Japan.

1.2. Characterization

^{1}H NMR spectra of the intermediate and final polymers were obtained at room temperature at 400 MHz on a JEOL JNM-ECS400 instrument. Molecular weight ($M_{n,SEC}$) and dispersity (M_w/M_n) of the polymers were measured by size exclusion chromatography (SEC) employing an Jasco high performance liquid chromatography system (PU-980 Intelligent HPLC pump, CO-965 Colum oven, RI-930 Intelligent RI detector, and Shodex DEGAS KT-16) using THF as the eluent at a flow rate of 1.0 mL min^{-1} at 40 °C. A series of polystyrene standards was used for the calculation of $M_{n,SEC}$ and dispersity M_w/M_n. The Fourier transform infrared spectroscopy (FT-IR) analysis was carried out using a PerkinElmer Frontier MIR spectrometer equipped with a single reflection diamond universal attenuated total reflection (ATR) accessory. XRD patterns were recorded using Cu Kα radiation (λ = 1.5418 Å) on a D2 Bruker diffractometer operating at 40 kV and 20 mA. The scanning electron

microscopic (SEM) (Hitachi TM-3000, Tokyo, Japan) images were obtained with a cold-field emission scanning electron microscope (HR-SEM) (Hitachi S-4800, Tokyo, Japan) equipped with energy-dispersive. UV-visible spectra were measured by spectrophotometer (Jasco V-730, Sendai, Japan). The PL spectra were measured by a Fluorolog-3 spectrofluorometer (Horiba Jobin Yvon, Paris, French), and the nanofiber mats were excited at wavelengths of 380 nm. The fluorescent images of $CsPbBr_3$@polymer fiber film were measured by Confocal Spectral Microscope Imaging System (Leica TCS SP5, Wetzlar, Germany). The water-resistant properties of the $CsPbBr_3$@polymer fiber film were observed through contact angle measurement (Phoenix 300 Touch, GAT Scientific, Suwon, Korea). The CIE color coordinates and CCT of the WLED spectra were measured by Spectroradimeter (PR670, Taipei, Taiwan) under ambient air conditions at 25 °C. The calibration method was standard sunset light, which correlated to a color temperature of 2856 K, to confirm instrument correctness. The TR-PL spectra were coupled to a spectrometer (Horiba iHR320) with a Hamamatsu C10910 streak camera and an M10913 slow single sweep unit. Temperature-dependent photoluminescence was measured with a pulsed diode-laser (PicoQuant LDH-D-C-375) at a repetition rate of 1 MHz.

1.3. Synthesis of the ethynyl end-terminated polyfluorene (PF)

In an argon-filled glovebox, 4-iodobenzyl alcohol (288 μL, 0.14 mmol, 0.5M mol L^{-1} stock solution in THF), $Pd_2(dba)_3$. $CHCl_3$ (44.66 mg, 0.04 mmol), *t*-Bu3P (633 μL, 0.32 mmol, as 0.5 mol L^{-1} stock solution in THF), and dry-THF (12 mL) were added to a vial and stirred for 30 minutes. A solution of PF monomer (7.15 ml, 3.45 mmol, 0.42M mol L^{-1} stock solution in THF), 18-crown-6 (9.44 mg, 35.75 mmol), and K_3PO_4 (22.77 mg, 107.25 mmol) were dissolved in a mix solvent of dry-THF (420 mL) and deionized water (54 mL), which has been purged with argon, and then cool to -20 ℃. After 30 minutes stirring, the Pd-initiator solution was introduced to monomer solution by a cannula to start the polymerization for 10 minutes. To terminate the polymerization, 12M HCl (10ml) was added to the reaction mixture and react for 30 minutes. After removing the solvent by evaporation, the residue was extracted by CH_2Cl_2 and brine. The organic layer was dried over $MgSO_4$ and purified by alumina. The residue was dilute by adding THF and drop to the cold toluene solution. The precipitate was collected by filtration and dried under reduced pressure to get PF-BnOH (953mg; yield: 85.10 %), a yellow powder. $M_{n,\ NMR}$ = 8,200 g mol^{-1}; M_w/M_n = 1.38. ^{1}H-NMR (400 MHz, $CDCl_3$): δ (ppm) 7.75-7.90 (m, Ar-H), 7.56-7.70 (m, Ar-H), 7.48 (d, Ar-H), 4.80 (d, -CH_2OH): 2.18 (br, -$CH_2(CH_2)_4CH_3$), 0.62-1.32 (m, -$CH_2(CH_2)_4CH_3$). The PF_{8k}-BnOH powder was dried overnight. In a three-necked flask, PF_{8k}-BnOH (900 mg, 0.11 mmol), EDC (105.20 mg, 0.55mmol), and DMAP (67.04 mg, 0.55 mmol) were dissolved in dry CH_2Cl_2 (100 mL). The reaction was deoxygenated by argon bubbling for 1 h, and then 5-hexynoic acid (59.86μL, 0.55mmol) was added to the solution. The reaction mixture was stirred for 24 h at room temperature, and the solvent was evaporated to dryness. The residue is dilute by adding THF and drop to the cold MeOH as a poor solvent to get alkyne PF_{8k} (857mg; yield: 95.2%), a yellow powder. $M_{n,\ NMR}$ = 8,300 g mol^{-1}; M_w/M_n = 1.43. ^{1}H-NMR (400 MHz, $CDCl_3$): δ (ppm) 7.74-7.88 (m, Ar-H), 7.58-7.76 (m, Ar-H), 7.48 (d, Ar-H), 5.24 (s, -CH_2-OC(=O)CH_2-), 2.55 (t, -OC(=O)CH_2-), 2.32 (m, CH_2C≡CH), 2.16 (br, -$CH_2(CH_2)_4CH_3$), 2.00 (t, -C≡CH), 1.92 (m,–CH_2CH_2C≡CH), 0.62-1.36 (m, -$CH_2(CH_2)_4CH_3$).

1.4. Synthesis of the azido-terminated poly (n-butyl acrylate) (PBA)

The ω bromo-terminated poly-(*n*-butyl acrylate) (PBA-Br) was prepared according to the reported paper. [27] *n*-Butyl acrylate (BA) (monomer) (18 mL, 0.126 mol, 126 eq) was purified by passing through a neutral Al_2O_3 column to remove out the inhibitor and mixed with ethyl 2-bromoiso butyrate (initiator) (0.146 mL, 0.001 mol, 1 eq), CuBr (catalyst) (57.135 mg, 0.003mol, 3 eq), and anhydrous toluene (2 mL) in Schlenk flask under argon atmosphere. After 1 hour, PMDETA (0.209 mL, 0.001mol, 1 eq) was added to flask. The atom transfer radical polymerization (ATRP) was performed in the oil bath at 70 ℃ for 2 hours followed by the terminal reaction through air bubbling. The mixture was purified by Al_2O_3 column chromatography and eluted with THF to remove out the catalyst. After that, the concentrated polymer solution was further purified by immersed in acetone

with a Spectra/Pro dialysis membrane (molecular weight cutoff 1.0 kDa) for 48 hours at room temperature. Finally, the polymer solution was evaporated to dryness, and a highly viscous and yellow PBA-Br liquid (8.91 g; yield: 55%) was obtained. Monomer conversion: 74%; $M_{n,\,NMR}$ = 11,800 g mol^{-1}; M_w/M_n = 1.10. ^{1}H NMR (400 MHz, $CDCl_3$): δ (ppm) 1.82-1.95 and 2.15-2.41 (br, polymer backbone); 3.83-4.10, 1.45-1.65, and 0.85-0.97 (br, butyl side chain).

The azidation was performed by following reported procedures [27] The obtained PBA-Br (8.63g, 0.72 mmole) and NaN_3 (233.76 mg, 3.60 mmole) were dissolved in DMF (15 mL) and stirred for 48 hours at 40 ℃. The excess NaN_3 in the solution was removed by passing through a silica gel column by DMF to get high viscous and yellow PBA-N_3 liquid. PBA_{12k}-Br conversion: 99%, PBA_{12k}-N_3: $M_{n,\,NMR}$ = 11,700 g mol^{-1}; M_w/M_n = 1.10. ^{1}H NMR (400 MHz, $CDCl_3$): δ (ppm) 1.82-1.95 and 2.15-2.41 (br, polymer backbone); 3.89-4.08, 1.45-1.65, and 0.85-0.97 (br, butyl side chain).

1.5. Synthesis of the PF-b-PBA block copolymer

The synthesis of the block copolymer (PF-*b*-PBA) (3) was performed by the click reaction between ethynyl-terminated PF (1) and azido-terminated PBA (2), as shown in **Scheme 1**. The alkyne PF_{8k} (200 mg, 25 μmol) was vacuum dried overnight before the click reaction and was introduced in a flask with CuI (23.8 mg, 125 μmol). The PBA_{12k}-N_3 (360 mg, 30 μmol), PMDETA (52.19 μL, 250 μmol), and anhydrous THF (7 mL) were placed in a 10 mL Schlenk flask. The mixture in the Schlenk flask was deoxygenated by argon bubbling for 1 h, which was then added to the flask under an argon atmosphere. The reaction flask was stirred for 42 h in an oil bath at 45 °C. The polymer solution was diluted by adding THF and purified by passing through a basic Al_2O_3 column to remove the catalyst. The residue was further concentrated by rotary evaporation and was precipitated in the cold mixed solvent (MeOH/DI water = 4/1 (v/v)) to remove the excess PBA homopolymer. After precipitation, the final product of PF_{8k}-b- PBA_{12k} (480 mg; yield: 85.7%) was obtained as a yellow sticky solid. $M_{n,\,NMR}$ = 19,100 g mol^{-1}; M_w/M_n = 1.43. ^{1}H NMR (400 MHz, $CDCl_3$): δ (ppm) 7.56-7.94 (m, Ar-H of PF backbone), 5.24 (s, -CH_2-OC(=O)CH_2-), 2.16 (br, -$CH_2(CH_2)_4CH_3$ of PF side chain), 0.62-0.87, 1.02-1.28 (m, -$CH_2(CH_2)_4CH_3$ of PF side chain), 1.82-1.95, 2.15-2.41 (br, PBA backbone), 3.88-4.12, and 0.85-0.97 (br, butyl side chain of PBA).

1.6. Synthesis of the $CsPbX_3$ (X = Cl, Br, I)

The $CsPbX_3$ (X = Cl, Br, I) was prepared by previous reported paper. [38] PbX_2 (0.188 mmol) and 1-octadecylen (ODE) (5 mL) were loaded into a 25-mL three-necked flask and degassed in a vacuum at 120°C for 1 h. Subsequently, dried oleylamine (0.5 mL) and oleic acid (OA) (0.5 mL) were injected into the solution at 120°C in an N_2 atmosphere. The mixture was heated to 170°C and a Csoleate solution (0.4 mL) was quickly injected into the mixture. After 5 s, the mixture was cooled. The nanoparticles (NCs) were extracted from the crude solution by centrifuging at 3500 rpm for 10 min. The precipitated particles were discarded, and the supernatant was redispersed in toluene to form the final solution.

1.7. Preparation of the electrospinning of $CsPbBr_3$ QDs/polymer nanofibers

The electrospinning nanofibers were prepared using a single-capillary spinneret. First, the $CsPbBr_3$ (100, 200, and 400 μL) was dissolved in CH_2Cl_2 (2mL). Second, the PF_{8k}-*b*-PBA_{12k} ($M_{n,\,SEC}$ = 21,400 g mol^{-1}) block copolymer (500 mg mL^{-1}) was added to the mixture and stirred overnight. The polymer solution was fed into a metallic needle using syringe pumps with a feed rate of 0.8-1.0 mL h^{-1} and the voltage was set at 10.2 kV during electrospinning. A piece of aluminum foil or quartz was placed 15 cm below the tip of the needle for 30-60 mins to collect the nanofibers.

References

1. Chou, S.-Y.; Ma, R.; Li, Y.; Zhao, F.; Tong, K.; Yu, Z.; Pei, Q. Transparent Perovskite Light-Emitting Touch-Responsive Device. *ACS Nano* **2017**, *11*, 11368-11375.
2. Lin, C.C.; Jiang, D.-H.; Kuo, C.-C.; Cho, C.-J.; Tsai, Y.-H.; Satoh, T.; Su, C. Water-resistant efficient stretchable perovskite-embedded fiber membranes for light-emitting diodes. *ACS Appli. Mater. Inter.* **2018**, *10*, 2210-2215.
3. Hsieh, H.-C.; Hung, C.-C.; Watanabe, K.; Chen, J.-Y.; Chiu, Y.-C.; Isono, T.; Chiang, Y.-C.; Reghu, R.R.; Satoh, T.; Chen, W.-C. Unraveling the stress effects on the optical properties of stretchable rod-coil polyfluorene-poly (n-butyl acrylate) block copolymer thin films. *J. Polym. Chem.* **2018**, *9*, 3820-3831.

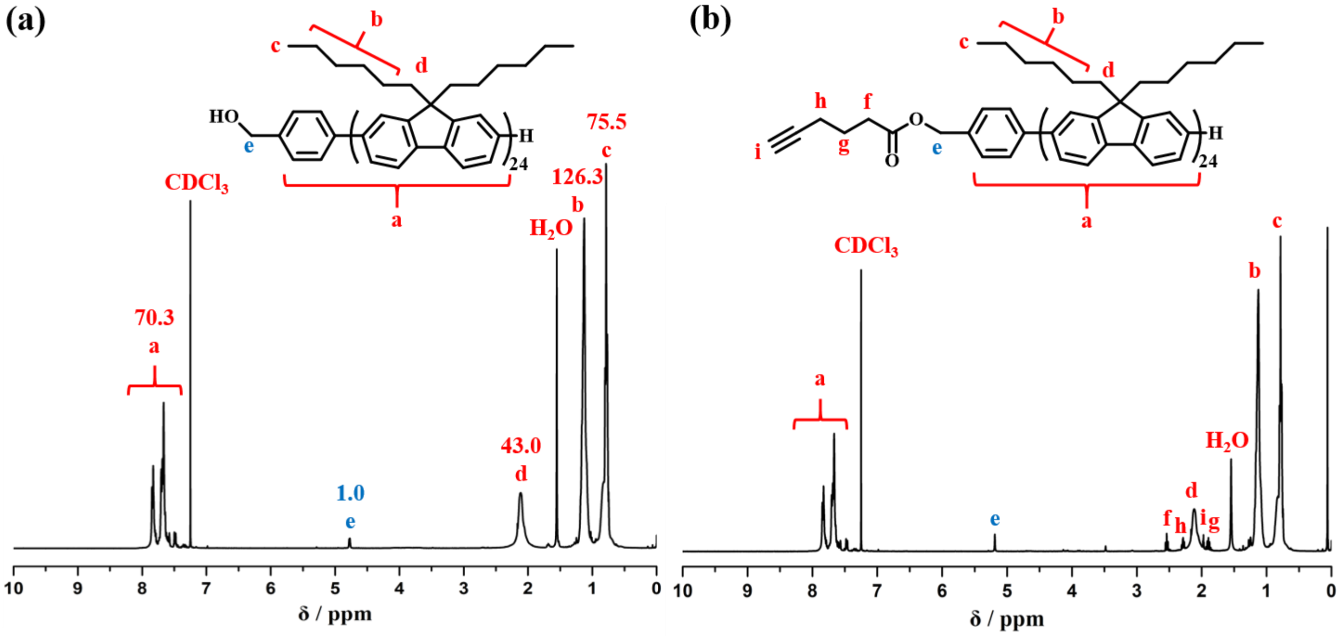

Figure S1. ^{1}H-NMR spectra of the (a) PF$_{8k}$-BnOH and (b) PF$_{8k}$-C≡CH in $CDCl_3$.

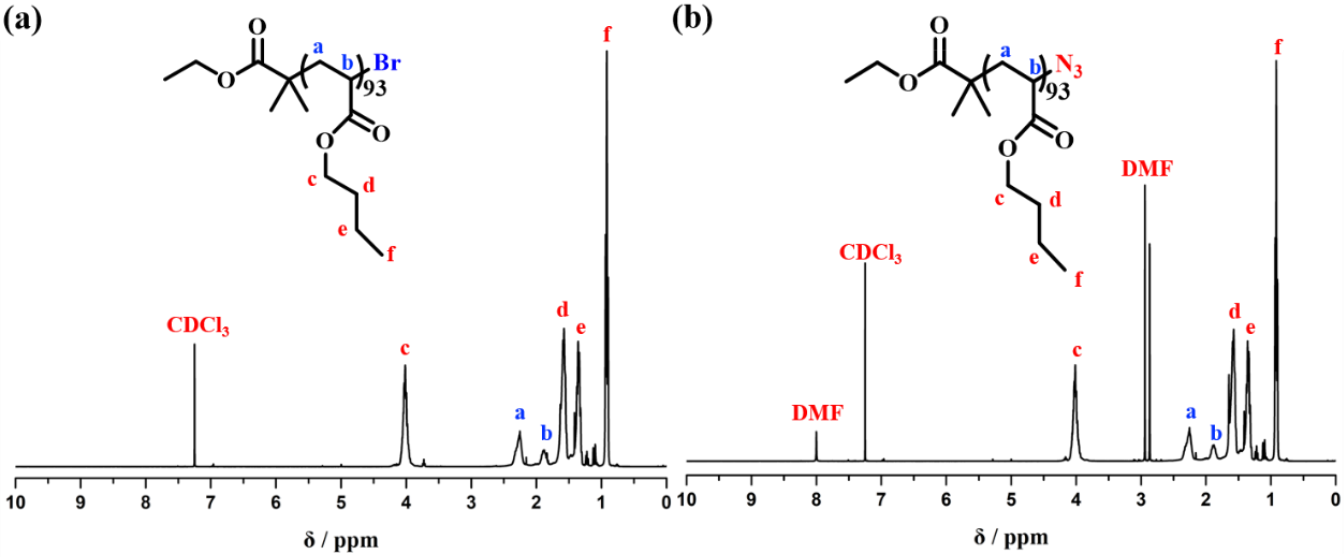

Figure S2. ^{1}H-NMR spectra of the (a) PBA$_{12k}$-Br$_3$ (b) PBA$_{12k}$-N$_3$ in $CDCl_3$.

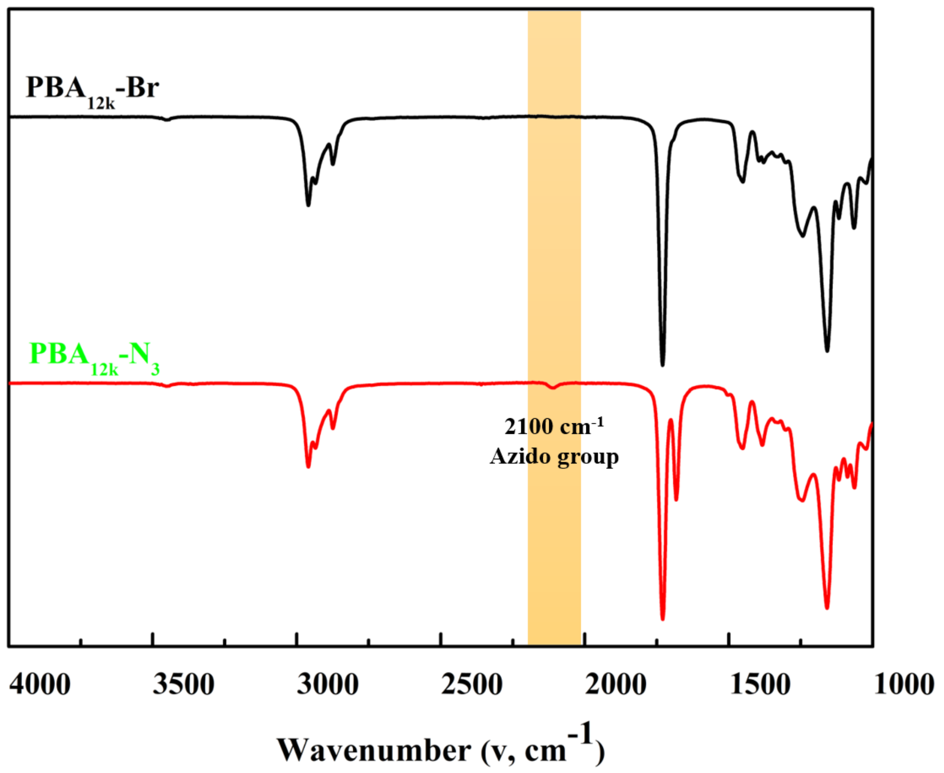

Figure S3. FTIR spectra of the PBA_{12k}-Br and PBA_{12k}-N_3.

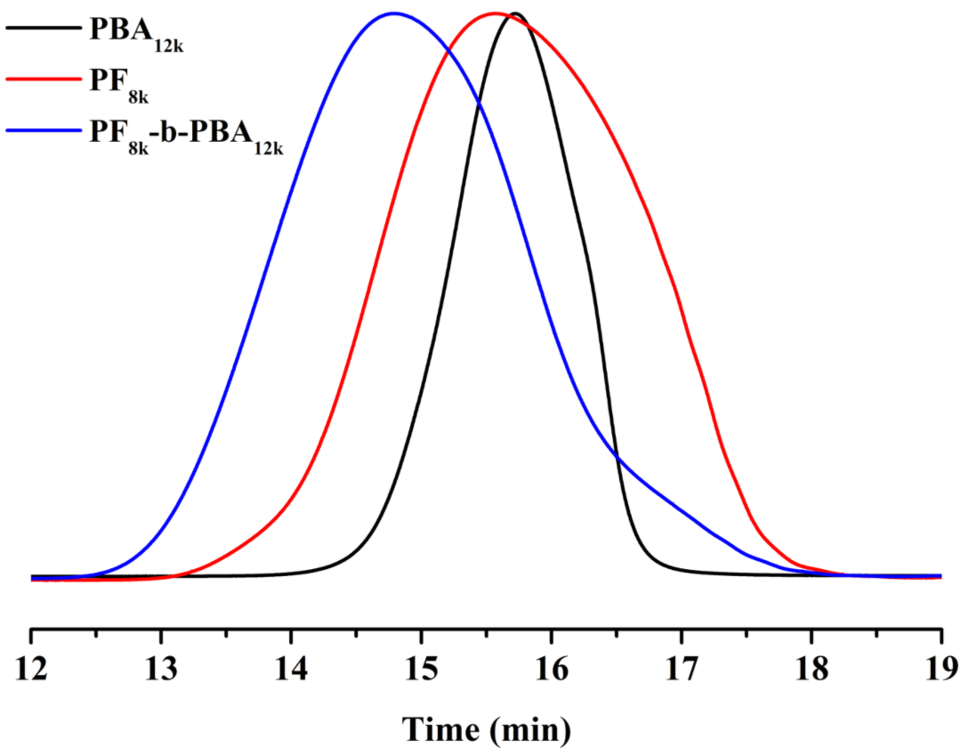

Figure S4. SEC profiles of the PF_{8k}, PBA_{12k}, and PF_{8k}-*b*-PBA_{12k} copolymers in THF eluent.

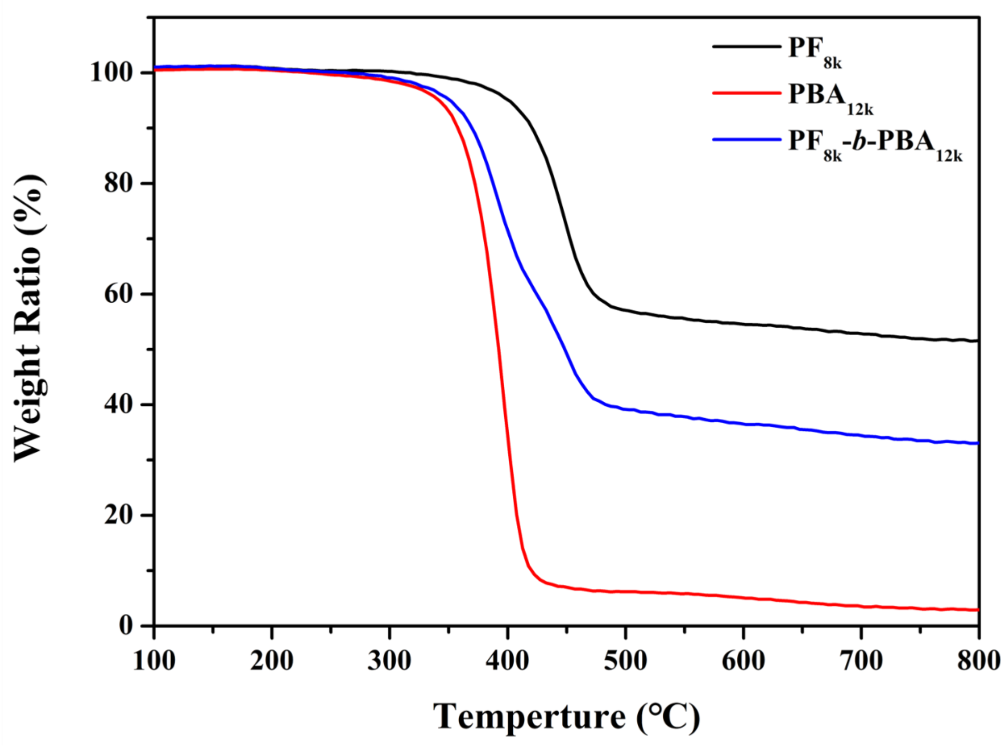

Figure S5. TGA curves of the PF_{8k}, PBA_{12k}-N_3, and PF_{8k}-*b*-PBA_{12k} copolymers.

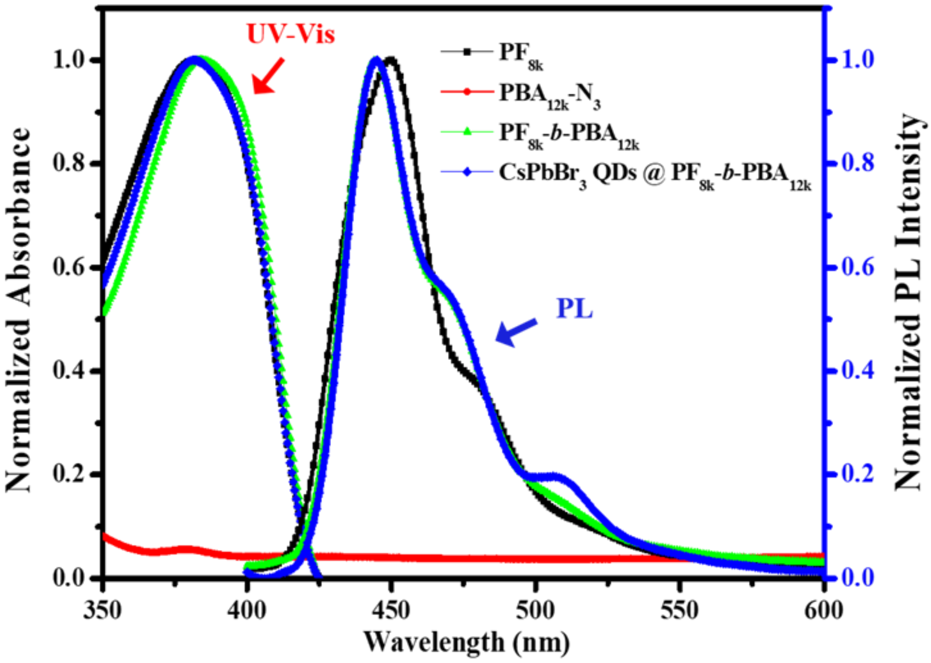

Figure S6. UV-vis absorption and PL emission spectrum of the PF_{8k}, PBA_{12k}-N_3, PF_{8k}-*b*-PBA_{12k}, and $CsPbBr_3$ QDs@PF_{8k}-*b*-PBA_{12k} solution.
